# Improving nurses’ knowledge, attitude, and performance in relation to ethical codes through group reflection strategy

**DOI:** 10.1186/s12912-021-00749-2

**Published:** 2021-11-06

**Authors:** Marzieh Momennasab, Marjan Ghanbari, Mozhgan Rivaz

**Affiliations:** 1 Department of Nursing, School of Nursing and Midwifery, Shiraz University of Medical Sciences, Shiraz, Iran; 2 School of Nursing and Midwifery, Shiraz University of Medical Sciences, Shiraz, Iran; 3 Community Based Psychiatric Care Research Center, Department of Nursing, School of Nursing and Midwifery, Shiraz University of Medical Sciences, Zand St., Namazee Sq, Shiraz, 7193613119 Iran

**Keywords:** Group reflection, Knowledge, Attitude, Performance, Nurses, Codes of ethics

## Abstract

**Background:**

The most basic responsibility of nurses that even precedes their therapeutic role is respect for professional ethics in providing clinical care. The present study was conducted to determine the effect of group reflection on the knowledge, attitude and performance of nurses in relation to ethical codes.

**Methods:**

The present blinded, before-after, educational trial was conducted on 86 nurses working at a general hospital in the south of Iran who were randomly divided into a intervention (*n* = 44) and a control (*n* = 42) group. Data were collected before and after the intervention using three tools, including a knowledge test, an attitude rating scale and a performance questionnaire. In the intervention group, the intervention given consisted of four sessions of group reflection, and the control group received a single lecture on ethical codes.

**Results:**

The mean changes in the nurses’ score of knowledge after the intervention compared to before differed significantly in both intervention and control groups (*P* < 0.001), but there was no significant difference between the two groups in terms of the mean changes in the score of knowledge (2.73 ± 3.45 in intervention group vs. 2.57 ± 3.36 in control group, *P* = 0.83). Although the mean score of attitude differed significantly between the intervention and control groups in the posttest (34.7 ± 8.44 in intervention group vs. 29.95 ± 9.09 in control group, *P* < 0.014), the two groups were not significantly different in terms of the mean changes in the score of attitude in relation to ethical codes before and after the intervention (*P* < 0.14). Moreover, the two groups were significantly different in terms of the mean changes in the scores of performance in the two stages (9.07 ± 16.84 in intervention group vs. 0.67 ± 20.01 in control group, *P* < 0.001).

**Conclusion:**

Group reflection can improve the knowledge, attitude and performance of nurses in relation to ethical codes. Although lectures can help improve nurses’ knowledge and attitude in this area, they have no significant effects on their performance.

**Trial registration:**

Iranian Registry of Clinical Trials (No: IRCT2016070317546N6, registration date: 10 October 2016), https://www.irct.ir/trial/16112

## Background

Ethical codes are ethical values in academic and clinical settings and a prominent aspect of the nursing profession [1]. These codes have been systematically developed in different countries throughout the world, and the Iranian Nursing Codes of Ethics was developed in 2010 with 12 values and 71 professional ethical codes in five domains and was completed and revised in 2012 [2].

Although Iran has developed systematic ethical codes for nurses, adherence to them in clinical settings has reportedly varied in different studies. Some have reported nurses’ performance in this area as unfavorable or semifavorable [3, 4] and others as desirable [5–7]. Performance is affected by the individual’s knowledge and attitude, especially in the area of ethics, which is influenced by the cultural and social context [8]. One of the reasons for nurses’ poor performance in the area of ethical codes is reportedly their lack of knowledge and inadequate training [4, 9, 10]. The notion that being a nurse enables the individual to have an ethical conduct without receiving any training is entirely unfounded [11]. The most important measure that should be taken in order to have capable and ethically-competent nurses who provide quality care is to establish and comply with the principles of professional performance through an emphasis on teaching ethical principles [12, 13]. Studies have shown that ethical education has a significant positive effect on the promotion of nurses’ ethical decision-making [14–16].

In Iran “nursing ethics” was added as a specialized course to BSc nursing program from 2014. Before that there was no any independent course in this regard in any levels of nursing education and nursing students had been learned ethical issues through hidden curriculum [17]. Because of this deficit in ethics education, nurses feel the need for training. In a study that investigated nursing ethics priorities on a national level in Iran, the results showed nursing ethics education is the second priority from the nurses’ viewpoint [18]. This result revealed the importance of effective ethics education in nursing.

Training nurses should actively develop their independence, critical thinking, open-mindedness and sensitivity to others [19, 20]. One way for active learning is through reflection. Reflection has been defined as a process of reviewing an experience in order to describe, analyze, and evaluate the performance [21]. This method is a reshaping of experience to improve learning and performance and is effective in increasing nurses’ awareness about and skills for clinical care and aims to improve their professional performance [22]. Reflection can also affect the individual’s attitude [23] and is particularly important in relation to ethical performance, which is also related to social and cultural conditions [20]. Group reflection is a method of reflection in which, through working in a small group, learners can share their reflections [24] for purposes of education that refers to the participation of groups of people in offering different perspectives on a given problem for better and clearer learning [25]. Thus, in addition to enabling the individual to focus more on and review his experiences, group reflection facilitates the use of different people’s views and perspectives [26, 27]. Studies have examined education through reflection and group reflection in different areas and have mostly demonstrated positive effects for these methods [25, 28, 29]. There are some models and frameworks to guide the reflective process. One of them that is used extensively in education and healthcare education.

is that developed by Graham Gibbs (1988) [24]. Gibbs’ Reflective Cycle is an extension of Kolb’s experiential learning cycle [30] and consist of six steps including description, feeling, evaluation, analysis, conclusion and action plan [31].

Given that most studies conducted on the degree of compliance with ethical codes in nursing have been descriptive, and very few have been interventional (especially in the cultural and social context of Iran), more studies are required to examine the effect of different educational methods on the knowledge, attitude and performance of nurses in this area. The present study was therefore conducted to determine the effect of teaching nursing ethical codes using group reflection on the knowledge, attitude and performance of nurses.

## Methods

The present single-blind, before-after, educational trial was conducted in a hospital affiliated to Shiraz University of Medical Sciences in the south of Iran.

Based on a study conducted by Shadfard in 2014 [32] and taking into account α = 0.05, β = 0.2, test power = 0.8 and potential withdrawal =10%, the sample size was determined as 45 per group. A total of 90 willing eligible nurses working at different wards of the described hospital were selected. The study inclusion criteria consisted of having a bachelor’s degree or higher in nursing, a minimum of 1 month of work experience and not having attended courses on ethical codes from 1 year ago. The study exclusion criteria consisted of more than two sessions of absence from the reflection training, not participating in the pretest or posttest and withdrawal from the project.

**Fig. 1:**
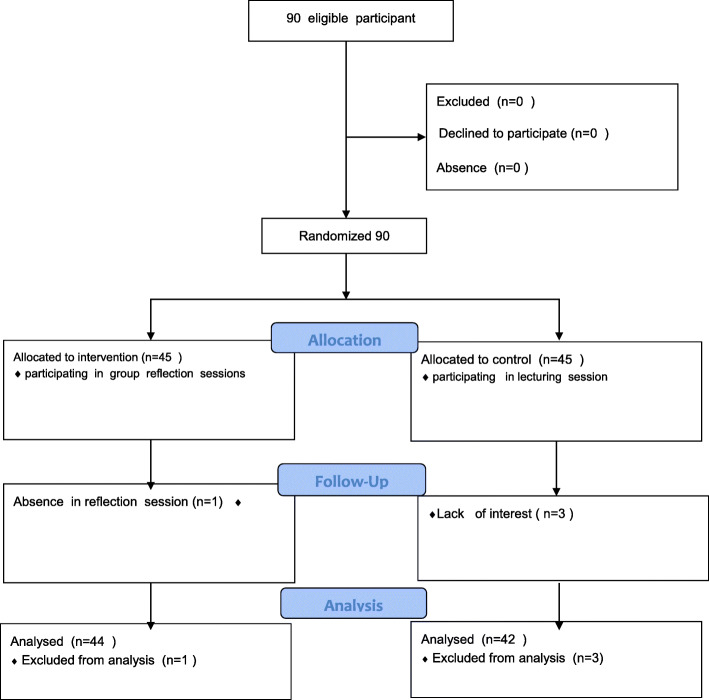
Flow of participants

To avoid the unwanted exchange of information between the groups during the study, multistage random sampling was performed. At first hospital wards were divided in to control and intervention groups by simple randomization. Participants from hemodialysis, CCU, emergency room, and medical wards were assigned in control group and who from ICU, NICU, operating room, pediatrics, and surgical wards were assigned in intervention group. The number of participants from each ward was determined using quotas and then nurses were selected by systematic random approach from the list of nurses working in each ward. The willing candidates entered the study until the group sizes reached 45. In the course of the study, one subject from the intervention group (for absence from the reflection sessions) and three from the control group (for unwillingness to continue their cooperation) were excluded. Ultimately, the data of 86 nurses (44 in the intervention group and 42 in the control group) were analyzed (Fig. 1).

A demographic questionnaire, a knowledge test, an attitude rating scale and a performance questionnaire were used for data collection.

### The knowledge test

This test was prepared by the researchers based on the available literature on ethical codes and Iranian Nursing Codes of Ethics. It contained seven true/false items and 12 multiple choice items. For each item, the correct answer was given one point and the other answers were scored zero, making the minimum score zero and the maximum 19. To evaluate the content validity of the test, a qualitative content validity assessment was carried out and the questionnaire was distributed among 12 nursing ethics experts and their comments on the content and quality of the designed items were taken. To examine the questionnaire’s reliability, the test-retest method was used. The questionnaire was completed by 21 nurses at the interval of 2 weeks. The Pearson correlation coefficient between the two tests was 0.9.

### The attitude rating scale

This scale was developed by the researchers based on the relevant literature and contained 17 items scored based on a four-point Likert scale. The items assessed nurses’ attitude on the necessity, significance and practicality of observing codes of ethics in providing patient care. Each item was scored from zero to three (from ‘totally agree’ =3 to ‘totally disagree’ =0), and the minimum score was zero and the maximum 51. To determine the content validity of the scale, the Waltz and Bausell (1983) Content Validity Index (CVI) according to the views expressed by 12 nursing professors was used [33]. For the reliability assessment, the test-retest method was performed and the scale was completed twice by 21 nurses at an interval of 2 weeks, and the Pearson correlation coefficient between the two tests was calculated as 0.94. Also, the Cronbach alpha coefficient of 0.76 showed the favorable internal consistency of the tool [33].

### The performance questionnaire

In the present study, ‘performance’ indicates nurses’ extent of observing ethical codes in providing clinical services. To this end, a questionnaire extracted from the Iranian Nursing Codes of Ethics (the section on Nurses and Practice with 23 codes) was used. This 26-item questionnaire was developed by Moemennsab et al. [6], and has a confirmed validity and reliability. Each item is scored from 0 for ‘never’ to 3 for ‘always’, and the minimum and maximum scores are zero and 78 [6]. In the present study, the content validity of the tool was assessed with the views expressed by 12 nursing ethics experts. For the reliability assessment, the questionnaire was completed twice by 21 nurses at an interval of 2 weeks, and the Pearson correlation coefficient between the two tests was calculated as 0.98. Nurses who participated in the process of assessing the reliability of the questionnaires were not included in the study samples.

The nurses in both the intervention and control groups were first briefed on the study objectives and methods in a session and their informed consents were obtained. They then completed the knowledge, attitude and performance questionnaires. The performance questionnaire was also completed for each nurse by the ward’s head-nurse, and the mean of the scores given by the nurses and the head-nurse was taken as the performance score of each subject. The intervention group was divided into five groups of nine, and 4 two-hour group reflection sessions were held for each group in the ward conference room. Each intervention group received two reflection sessions weekly. In each session, two scenarios about observing ethical codes were discussed and reflected on. The scenarios were based on the researchers’ experiences and the Nursing Codes of Ethics and used the available resources and were approved by 12 professors at the school of nursing. These scenarios included clinically-tangible issues with which the personnel were faced on a daily basis and included topics such as the importance of obtaining informed consent, respect for the privacy of the patients and their confidentiality, preserving their right to autonomy and decision-making, respect for the patients’ personal beliefs, preserving their right to choose to continue the treatment and choose a nurse, the respectful treatment of the patients and other colleagues, and refraining from the commercial promotion of any particular products. Seven questions were posed at the end of each scenario that debated and assessed the subjects’ understandings, feelings, views and perspectives and potential decisions.

The reflection sessions were guided by the group leader (researcher) according to the Gibbs model. Gibbs’ reflective circle involves description, feeling, evaluation, analysis, conclusion and action plan [31]. In line with this model, questions were asked about each scenario and put to debate. All the principles of group dynamics were fully observed. The control group also received a two-hour lecture by the researcher on issues related to nursing codes of ethics along with a Q&A and a slide show. The posttest was held in the intervention and control groups 1 month after the last session of group reflection. Moreover, the nurses’ performance was assessed by their ward head-nurse, and the mean score of their performance was determined. To blind the study, the distribution and collection of the questionnaires and the statistical analysis of their data were performed by people blinded to the grouping. By the end of the educational intervention and after the posttest, all the participants in both groups were given the discussed scenarios and an educational booklet and a book on nursing ethics.

#### Ethical considerations

This study was approved by the research ethics committee of Shiraz University of Medical Sciences (No: IR.SUMS.REC.1395.55). It was also registered in the Iranian Registry of Clinical Trials (No: IRCT2016070317546N6). After receiving explanations about the study, all participants signed a written consent form. They were assured that rejecting participation in the study would have no effect on their professional status and their data would be keptconfidential and anonymous.

#### Statistical analysis

Data were analyzed by the software SPSS version 21.0 for Windowssoftware package. Descriptive statistics were used to describe the characteristics ofnurses and for comparisons between groups, chi-square, and independent-samples t-test were used. For all tests, results were considered statistically significant at *p* < 0.05.

## Results

The majority of the participants (82.6%) were female, married (64%) and had a bachelor’s degree in nursing (97.7%). The nurses were aged 23 to 47 with a mean (SD) of 30.55 (5.06) years. The mean (SD) of the nurses’ work experience was 6.5 (8.74) years in the intervention group and 7 (8.74) years in the control group. There were no significant differences between the two groups of nurses in terms of age, gender, marital status, academic qualifications or work experience (Table 1).

**Table 1:** Demographic characteristics of subjects in the two intervention and control groups

Characteristics	All subjects	Intervention)***n*** = 44)	Control) ***n*** = 44)	***P***-value
**Age (years)**				
Mean (SD)	*30.55 (5.06)*	*30.15 (4.96)*	*30.95 (5.17)*	*0.470* ^a^
**Years of work (years)**				
Mean (SD)	*6.75 (8.74)*	*6.5 (8.74)*	*7 (8.74)*	*0.623* ^a^
**Sex n (%)**				
male	15 *(17.5*)	*10 (22.7)*	*5 (11.9)*	
female	*71 (82.5)*	*34 (77.3)*	*37 (88.1)*	*0.183* ^b^
**n (%) Marital status**				
single	*31 (36.1)*	*19 (43.2)*	*12 (28.6)*	
married	*55 (63.9)*	*25 (56.8)*	*30 (71.4)*	*0.185* ^b^
**Educational level n (%)**				
baccalaureate	*84 (6.97)*	*43 (97.7)*	*41 (97.6)*	
**postgraduate**	***2 (2.4)***	***1 (2.3)***	***1 (2.4)***	*1.000* ^b^

^a^Chi-square test

^b^Independent t-test

There was no significant difference between the intervention and control groups in terms of the mean score of knowledge before the intervention, but after the intervention, the mean score of knowledge increased in both groups, and the independent t-test showed significant differences between the two groups after the intervention (*P* < 0.001). The independent t-test showed no significant differences between the two groups in terms of the mean changes in the score of knowledge (2.73 ± 3.45 in intervention group vs. 2.57 ± 3.36 in control group, *P* = 0.83). In other words, teaching ethical codes using group reflection and lecture increased the nurses’ ethical knowledge (Table 2).

**Table 2:** Comparison of the variable scores among two intervention and control groups

Variables	Group	Pretest mean	Post-test mean	Mean change	***P***-value^a^
**Knowledge**	intervention	10.50 (2.73)	13.22 (3)	2.73 (3.45)	0.001
	control	10.23 (2.22)	12.80 (3.11)	2.57 (3.36)	0.001
	P-value^b^	0.628	0.578	0.83	
**Attitude**	intervention	29.63 (7.49)	34.70 (8.44)	5.06 (8.99)	0.001
	control	27.78 (5.49)	29.95 (9.09)	2.17 (9.15)	0.133
	P-value^b^	0.197	0.014	0.14	
**Performance**	intervention	35.79 (9.49)	45.46 (11.39)	9.07 (16.84)	0.001
	control	35.20 (7.28)	38.78 (11.31)	0.67 (20.018)	0.077
	**P-value**^b^	**0.747**	**0.008**	**0.038**	

^a^paired t-test

^b^Independent t- test

The independent t-test showed no significant differences between the two groups in terms of the mean score of attitude before the intervention, but this difference was significant after the intervention (34.7 ± 8.44 in intervention group vs. 29.95 ± 9.09 in control group, *P* < 0.014). In the intervention group, the mean score of attitude showed a significant difference in the posttest compared to the pretest (*P* = 0.001), but no such significant difference was observed in the control group (*P* = 0.133; Table 2). Nevertheless, no significant differences were observed between the two groups in terms of the mean changes in the scores of attitude before and after the intervention (*P* = 0.14; Table 2).

The majority of the nurses in the intervention group (84%) believed, in the pretest stage, that observing ethical codes slowed them down, but in the posttest, only 21% of them still held this belief. In the pretest, 33 and 40% of the nurses totally agreed with the items “Observing ethical codes increases patient satisfaction” and “Observing ethical codes leads to professional improvement”, which increased to 72 and 76% in the posttest.

No significant differences were observed between the two groups before the intervention in terms of the mean score of performance, but the paired t-test showed a significant difference in the intervention group before and after the intervention (*P* = 0.001), while this difference was not significant in the control group (*P* = 0.077). The two groups were also significantly different in terms of the mean changes in their performance scores before and after intervention (9.07 ± 16.84 in intervention group vs. 0.67 ± 20.018 in control group, *P* = 0.038; Table 2). No significant relationships were observed in the present study between the changes in the scores of knowledge, attitude and performance and any of the personal demographic characteristics.

## Discussion

The present findings showed that both group reflection and lecture are effective strategies for promoting nurses’ knowledge of ethical codes. The results of other studies also confirm the positive effects of education through different educational strategies on nurses’ knowledge of ethics. In accordance with present study the results of some other studies showed lecture have had similar cognitive learning outcomes in ethics education to some student-centered learning strategies such as problem- based [34, 35] or team- based learning [36]. The results obtained by Farid et al. (2011) showed that teaching ethical principles by different methods can increase nurses’ knowledge of ethical issues and can improve their ethical judgment [37]. Many studies have shown that teaching ethics has a significant positive effect on nurses’ ethical decision-making [14, 38, 39]. Accordingly, Cusveller (2012) considers ethical knowledge one of the factors affecting nurses’ participation in solving ethical challenges [40].

Teaching ethical codes through group reflection was also effective in improving the nurses’ attitude. The mean changes in the scores of attitude before and after the intervention did not differ significantly between the two groups, which shows that teaching through lectures has also been able to improve nurses’ attitude. The results of another study conducted in Iran showed that teaching ethical codes changes the attitude of nurses toward these codes and consequently strengthens their commitment to ethics in providing care [41]. The results obtained by McCrink (2011) revealed that teaching ethics to nursing students deepens their attitude toward ethical issues [42]. Nurses with a poor attitude toward ethical issues often have a poor knowledge about the subject [43]. Meanwhile, reinforcing nurses’ attitude toward ethical codes is regarded as a key predicting factor in the nurses’ commitment to ethical care [42]. If the aim is to change attitudes, various effective strategies should be used to pave the way for raising awareness and knowledge and consequently improving attitude, so that the best performance and conduct can be achieved [44]. The results of one study showed that different teaching methods have different effects on people’s attitude, and compared to lectures and role-play, group discussion has a more significant effect [45]. The reason for the effect of lecturing on nurses’ attitude in the present study may be that the nurses in this center had never been systematically exposed to the codes of nursing ethics and professional ethics, and this intervention was effectively their first exposure and thus managed to promote their knowledge and attitude in this area.

This educational course on ethical codes using group reflection improved the nurses’ performance in the intervention group. The results of other studies also show the effectiveness of educational courses in improving nurses’ ethical performance [46–49]. The results obtained in a study conducted by Jafarimanesh et al. showed that, although the mean score of compliance with ethical codes in nursing students who had attended ethical training courses was higher compared to the control group, the difference between them was not statistically significant [50]. The results of another study showed that, although teaching ethics to nursing students enabled them to identify ethical violations in the workplace, this training had no effect on their ethical sensitivity [48]. The disparity of findings observed may owe to the different teaching methods used.

According to some studies, teaching ethics in a group setting is more effective than the use of other methods [26, 47, 49, 51–53]. In the present study, teaching by group reflection had positive effects on the knowledge, attitude and performance of nurses in relation to the codes of ethics. Group reflection is a student-centered teaching method that has been found beneficial to the development of critical thinking and the improvement of ethical decision-making skills [26, 27]. Using these innovative methods increases participants’ thinking skills and allows them to convey and reflect their experiences and have a more in-depth learning beyond the existing cultural and preferential barriers [54]. By enabling reflection on past experiences and performances, group reflection facilitates internal judgment. Furthermore, by observing other people’s points of view, people can think about and reflect on problems from different angles. In one study, Callister et al. (2009) concluded that guided reflection effectively reinforces students’ critical thinking and ethical reasoning [26]. The results obtained by Kalaitzidis et al. (2012) showed that critical thinking and problem-solving skills are strengthened in nursing students who learn ethical codes through discussions and debates about simulated scenarios and talking about other people’s experiences [55]. These benefits of group reflection make it a useful and applicable teaching- learning strategy for ethical education in nursing, especially for improving nurses’ ethical performance.

In the present study, the lecture method was also able to improve the learners’ knowledge and attitude, but their performance remained unchanged. Since the goal of teaching is to improve awareness, change attitude and thereby behavior, active teaching methods with group participation that enable a longer-lasting learning are essential [56, 57]. It can be concluded that teaching ethical codes by group reflection is an efficient and economical method for teaching ethics to nurses that can improve their knowledge, attitude and performance.

In the present study, no significant relationships were observed between participants’ personal demographic and professional characteristics and the changes in their scores of knowledge, attitude and performance, which means that participants’ characteristics did not affect the study findings. Other studies have reported different results regarding the relationship between participants’ demographic variables and their ethical knowledge and attitude [41, 46, 58].

One of the limitations of this study was that the study setting was confined to only one health center, which undermines the generalizability of the results. Future studies are therefore recommended to be conducted on larger groups of nurses from a greater diversity of health centers. Although attempts were made to select the intervention and control groups from different wards, and although the participants were asked not to exchange information with each other, such exchange may have happened in some cases, and this limitation was beyond the researcher’s control.

## Conclusion

The present study showed that group reflection on the ethical codes of nursing improves nurses’ knowledge, attitude and performance, and this method was found to be more effective than traditional teaching methods such as lectures. This active teaching method, which can be implemented with minimal equipment, can be used to improve nurses’ commitment to nursing codes of ethics. The results of this study guide nursing managers for increasing nurses’ ethical performance using group reflection in continuous education courses. Nonetheless, traditional methods such as lectures can also be effective to a degree in cases where group reflection is not possible, since these methods also increase nurses’ knowledge about ethical issues.

## Data Availability

Data available by contacting the corresponding author.
